# Mucus interaction to improve gastrointestinal retention and pharmacokinetics of orally administered nano-drug delivery systems

**DOI:** 10.1186/s12951-022-01539-x

**Published:** 2022-08-06

**Authors:** Deepak A. Subramanian, Robert Langer, Giovanni Traverso

**Affiliations:** 1 Department of Chemical Engineering and David H. Koch Institute for Integrative Cancer Research, Massachusetts Institute of Technology, Cambridge, MA USA; 2 Department of Mechanical Engineering, Massachusetts Institute of Technology, Cambridge, MA USA; 3 Division of Gastroenterology, Brigham and Women’s Hospital, Harvard Medical School, Boston, MA USA

**Keywords:** Oral delivery, Gastrointestinal tract, Mucus, Mucoadhesive, Mucus penetration

## Abstract

Oral delivery of therapeutics is the preferred route of administration due to ease of administration which is associated with greater patient medication adherence. One major barrier to oral delivery and intestinal absorption is rapid clearance of the drug and the drug delivery system from the gastrointestinal (GI) tract. To address this issue, researchers have investigated using GI mucus to help maximize the pharmacokinetics of the therapeutic; while mucus can act as a barrier to effective oral delivery, it can also be used as an anchoring mechanism to improve intestinal residence. Nano-drug delivery systems that use materials which can interact with the mucus layers in the GI tract can enable longer residence time, improving the efficacy of oral drug delivery. This review examines the properties and function of mucus in the GI tract, as well as diseases that alter mucus. Three broad classes of mucus-interacting systems are discussed: mucoadhesive, mucus-penetrating, and mucolytic drug delivery systems. For each class of system, the basis for mucus interaction is presented, and examples of materials that inform the development of these systems are discussed and reviewed. Finally, a list of FDA-approved mucoadhesive, mucus-penetrating, and mucolytic drug delivery systems is reviewed. In summary, this review highlights the progress made in developing mucus-interacting systems, both at a research-scale and commercial-scale level, and describes the theoretical basis for each type of system.

## Introduction

Oral delivery is preferred over parenteral delivery because it is easier to administer, it is less invasive (thus lowering the sterility requirements), and it is less painful. This is associated with increases in patient adherence, which translates into increases in the effectiveness of the treatment [1]. Oral delivery is routinely utilized for small molecules; however, the biology of the gastrointestinal (GI) tract inhibits effective oral delivery of large drug depots (1–10 g) and macromolecular biologic drugs (e.g., peptides, proteins). Obstacles include degradation of biologic drugs, rapid GI transit time, and inefficient drug transfer from the GI tract to the bloodstream, all of which ultimately lead to poor drug bioavailability [2–7]. Overcoming these obstacles could significantly reduce pill burden for chronic drug regimens and ultimately increase therapeutic efficacy [8].

Oral drug delivery relies on the absorption of drugs from the GI tract to the bloodstream. However, there are various pH extremes and enzymes including proteases, amylases, and nucleases as well as bacteria throughout the GI tract that can degrade drugs including biologic drugs. Orally ingested materials transit through the whole GI tract in about 24 h, which reduces the amount of drug that can be delivered at any location, potentially lowering drug bioavailability and efficacy, and requiring frequent dosing for long-term drug regimens. By encapsulating drugs inside nanoparticles, release can be controlled and the drug cargo protected [9–12]. Methods to increase retention or delay elimination of orally delivered nanoparticle drug delivery carriers in the GI tract have been studied in order to improve drug pharmacokinetics [13–17].

Mucus is ubiquitous throughout the GI tract and can be used to prolong drug carrier residence time. Mucus-interacting mechanisms enable increased residence time of drug delivery carriers in the GI tract by allowing therapeutics either to adhere to the surface of the mucus layers or move through the mucus layer and bind to the surface of the epithelial cells. This review will discuss the properties of GI mucus that make it attractive for GI-retentive strategies and will then cover the three major types of mucus-interacting nanoparticle systems that have been developed for prolonged drug delivery after oral ingestion: mucoadhesive, mucus-penetrating, and mucolytic.

## Mucus in the GI tract—properties and function

### Background

**Fig. 1:**
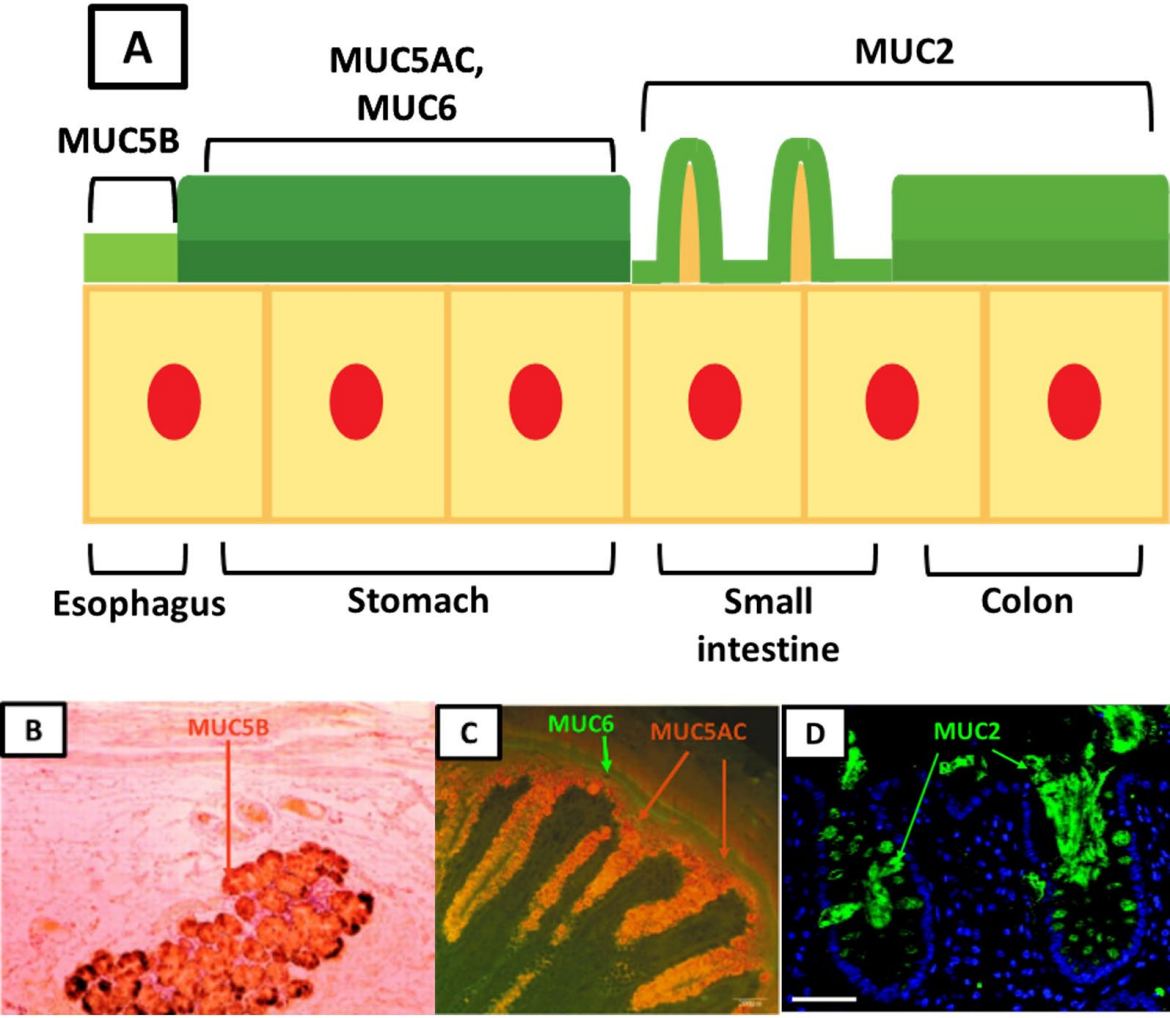
Schematic of the mucus layers in the GI tract as a whole (**A**), as well as tissue histology of the mucin MUC5B in the submucosal glands of the esophagus (**B**) as shown by Arul et al. [23], the mucins MUC5AC/MUC6 in the stomach (**C**) as shown by Ho et al*.* [24], and the mucin MUC2 in the small intestine (**D**) as shown by Gustafsson et al*.* [25]. The schematic (**A**) shows the esophagus (left), stomach (left middle), small intestine (right middle), and colon (right). The esophagus contains a thin layer of mucin MUC5B. The stomach contains two layers of mucin MUC5AC: a thin layer firmly attached to the epithelium and a thicker, loosely attached layer above. This outer layer also contains “bands” of mucin MUC6. The small intestine and colon both contain the mucin MUC2, but the small intestine only contains a thin, loosely bound layer. The colon is organized similarly to the stomach, with a thin, firmly attached layer and a thicker, loosely attached layer above

The mucus layers of the human GI tract are primarily composed of mucin proteins, which are generally clustered into highly glycosylated and non-glycosylated mucin domains [18]. There are two types of mucins: transmembrane mucins, which are found in the cell membrane and are chiefly located on the apical side of epithelial cells, and gel-forming mucins secreted by mucus-producing cells [18]. The mucus layers are composed of gel-forming mucins which are produced at specific regions of the human GI tract. Mucus layers are found throughout the length of the human GI tract and are composed of unique mucins: salivary gland and esophageal mucus contain the gel-forming mucin MUC5B [19], stomach mucus contains the gel-forming mucins MUC5AC and MUC6 [20], mucus in the small intestine primarily contains the gel-forming mucin MUC2 [21], and colon mucus contains the gel-forming mucin MUC2 [22] (Fig. 1).

The mucus layers present in the GI tract serve to protect the underlying epithelial surface from harmful substances and pathogens by impeding the diffusion of pathogens towards the epithelium [26]. The mucus layer in the small intestine contains a high concentration of antibacterial peptides and proteins, which remove bacteria that diffuse through the upper levels of the mucus layer [27–29]. This protective role is especially important in the small intestine, since the risk of infection in this region is greater due to the loose layer of MUC2 compared to the multi-layered mucus structure (containing a firmly attached bottom layer and a loosely attached upper layer) in the stomach and colon [30]. Unfortunately, this protective function also reduces the diffusion of drugs (hydrophilic and lipophilic) towards the epithelium [31], meaning that mucus also acts as a “barrier” that must be overcome in order to achieve successful oral drug delivery.

### Composition, structure, and material interactions

Mucus layers are composed of long, highly glycosylated protein chains that contain “PTS” domains, which consist primarily of the amino acids—proline, threonine, and serine. The PTS domains are glycosylated through glycan linkages to the threonine and serine amino acids, and these glycans contain negatively charged sialic acid and sulfate groups on their ends [32, 33]. This structure ultimately contributes to the highly negative charge density present in the PTS domains in mucins [33]. Before secretion, the negatively charged sialic acid groups attract cationic H^+^ and Ca^2+^ ions, which crosslink the glycans to form a condensed structure; however, these ions diffuse away from the mucin structure immediately after the mucin is secreted, allowing for rapid expansion of the mucin into a gel-like structure via charge repulsion [34]. This process allows the layer to maintain its integrity even as mucus is continually produced and cleared, which mitigates pathogen invasion into the epithelial cell layer.

**Fig. 2:**
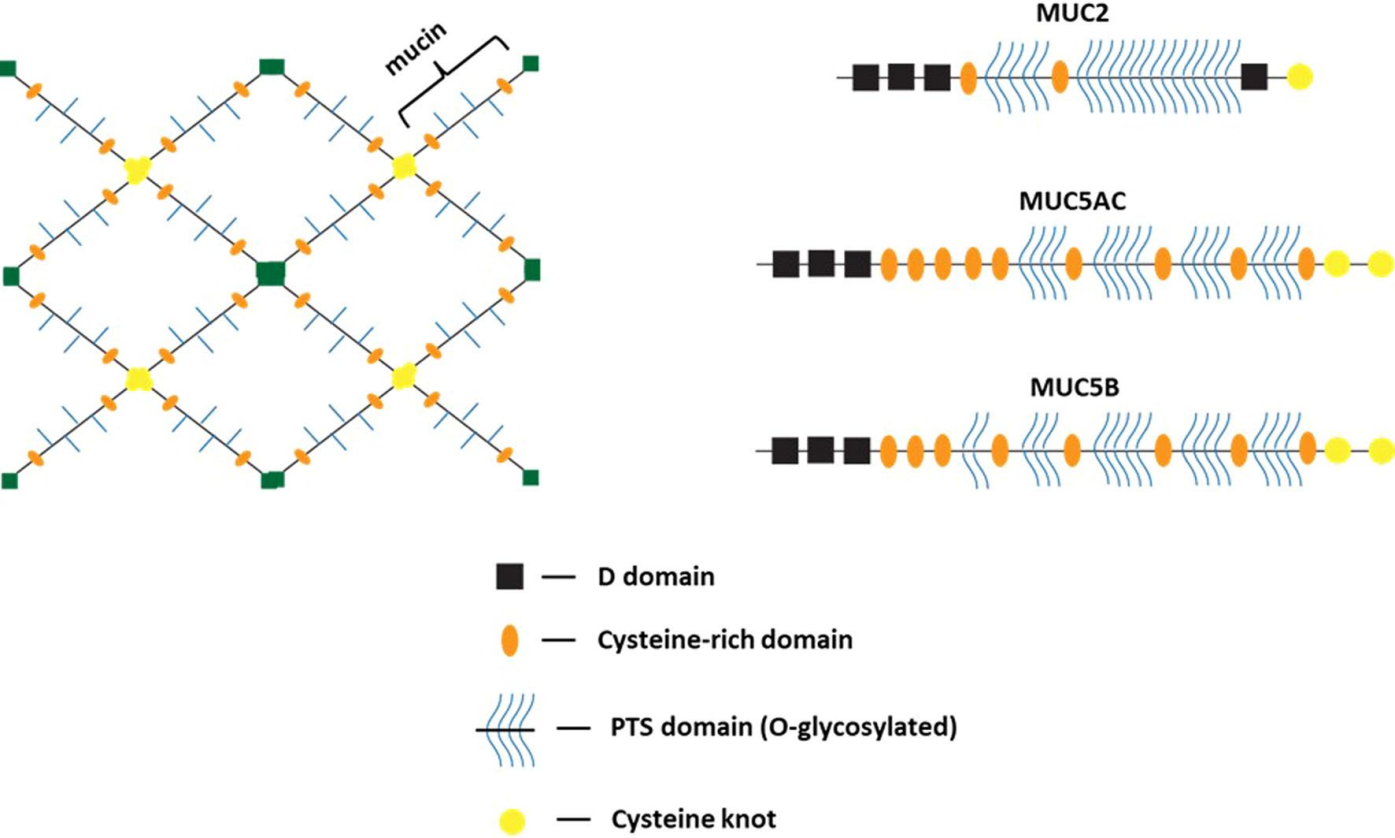
Schematic showing a crosslinked mucin structure. The image on the left shows the multimeric structure of the gel, in which individual mucin chains are connected via either their N-terminal D domains (in the trans-Golgi compartments of goblet cells) or through disulfide bonds formed between the cysteine knot regions. The image on the right shows the general structure of each of the three major GI mucins MUC2, MUC5AC, and MUC5B, with the indicated regions shown below in the legend. Figure adapted from Moran et al*.* [40]

The glycosylation state of the mucin proteins depends on the region of the protein. The PTS domains in secreted mucins are 25–200 nm in length along the peptide backbone, and the glycans that bind to the PTS domains generally form a “bottle-brush” structure [33]. These glycans can consist of up to 20 sugar monomers and can extend up to ~ 5 nm from the peptide backbone of the mucin [33]. However, the addition of *O*-linked glycans such as *N*-acetylgalactosamine (GalNAc) and the repulsive interactions between negatively charged sialic acid residues causes the side chain extensions of the PTS domains to extend up to ~ 15 nm [35]. With a larger persistence length, the likelihood of entanglement between mucin chains increases due to the greater number of possible interactions between the chains. The viscoelasticity of the mucus increases as a result, which reduces the ability of pathogens to permeate the mucus layers. The second type of domain within the mucin layers is the non-glycosylated, cysteine-rich domain [36]; the cysteine residues facilitate disulfide bond formation with other mucin chains or sulfur-containing compounds, resulting in greater interchain interactions. Because of the presence of the glycans [37], cysteine residues [38], and negatively charged sialic acid groups [39], mucins crosslink and form a mesh-like gel structure (Fig. 2).

Mucus gels are dynamic and do not exhibit a consistent mesh size throughout the mucus layer or over time [33]. In addition, the mesh-like structure has different properties than those expected from biochemical analysis, with fresh mucus having a mucin fiber thickness of 30–100 nm [41]. The expected thickness of mucus based on the length of the glycans is 3–10 nm [33]. Experiments on ovulatory cervical mucin suggest that the mucin pore size in the cervix could range from 20–200 nm and would be around 100 nm on average [42].

Some hypothesize that the mucin pore size in the small intestine could be as large as 211 nm based on studies with porcine intestinal tissue [43]. However, experimental data suggest that this pore size is likely smaller than this reported estimate. Ensign et al., based on their experimental data, conclude that murine colonic mucus has a pore size small enough to physically trap 200 nm nanoparticles and severely restrict the diffusion of 100 nm nanoparticles, while murine small intestinal mucus has a larger pore size based on reduced inhibition of 200 nm nanoparticles [44]. Abdulkarim et al. found that the pore size in porcine intestinal mucus was heterogeneous, but suggested that ~ 50% of pores within intestinal mucus had a size smaller than 200 nm [45]. Celli et al. suggested that the pore size of porcine gastric mucus at a pH of 2 is ~ 270 nm [46].

The pore size is also affected by environmental factors such as mucus concentration, pH, and change in [Ca^2+^], based on experiments using porcine and murine intestinal mucus [47–50]. Particle diffusion (even for smaller nanoparticles with size ~ 100 nm) is also affected by intermolecular interactions between the nanoparticles and the mucus layer, based on experiments with porcine jejunal mucus [51]. Dietary factors such as the presence of β-glucan have been correlated with changes in porcine intestinal mucus permeability and mucus pore size [52]. Others have shown a correlation between mucus pore size and certain disease states; one example is the apparent increase in murine mucus pore size in the presence of ulcerative colitis [53]. Another factor that influences mucus structure and composition is age; while GI mucus production has been shown to increase in newborn humans [54], the number of mucus-secreting goblet cells in humans has been shown to decrease with age [55, 56], and gastric mucus secretion in humans has been shown to decrease over time [57].

Materials can form monovalent or polyvalent bonds with mucin fibers, which affect their ability to diffuse through the mucin network [58]. Materials that interact with nonpolar solvents such as oil diffuse more slowly through mucus than through water, which suggests the presence of hydrophobic domains within the mucin structure [59]. The polyvalent, higher-affinity bonds present in some macromolecules (> 1000 Da) can impede diffusion through mucin [60]. In addition, cationic molecules such as chitosan can form tight polyvalent bonds with the negatively charged glycan groups of mucin, which also improves adhesion [60].

### Mucus turnover

Mucins are constantly being produced and shed throughout the GI tract. The thickness of the mucus layer, which is thickest in the stomach and colon and thinnest in the small intestine, is dependent on the balance between mucus secretion and mucus turnover [61]. In general, the rate of mucus turnover is dependent on GI digestion activity; the upper layer, which is distal to the epithelium, is more loosely attached and is more sensitive to digestive conditions such as the movement of digested materials and chyme [62]. In areas where there are higher levels of gastric motility and proteolytic activity, the mucus turnover rate is higher due to enzymatic or mechanical degradation of the mucus and subsequent digestion and clearance [62]. Mucus turnover is important because it facilitates the removal and excretion of accumulated foreign materials and pathogens from the body. Table 1 shows the turnover rates for different portions of the GI tract.

**Table 1:** Mucus turnover in different regions of the GI tract

Region	Turnover rate	References
Stomach	~ 5–6 h	[63]
Small intestine	6 h (villi), 7 h (crypt)	[21]
Colon	~ 1 h (inner layer)	[64]

### Mucus layers in disease states

The mucus layers in the GI tract change during disease with significant consequences [65]. Two important pathological changes that are correlated with GI disease states are over- and under-production of mucins, which lead to a thicker and thinner mucus layer, respectively. These changes disrupt the GI homeostasis and can affect mucus layer function. An overproduction of mucins can obstruct the digestive tract; underproduction of mucins can enable bacteria to contact the intestinal epithelium which can trigger inflammation. Overproduction of mucins is observed in certain types of mucinous carcinomas [66], while underproduction of mucins is found in ulcerative colitis [67, 68]. Other disease states, such as adenocarcinomas of the small intestine and colon, are correlated with changes in mucin glycosylation, which can alter the microenvironment and support tumor growth [69].

Mucinous carcinomas, which account for about 6–19% of all types of colorectal cancers, are intestinal cancers in which mucins make up at least 50% of the tumor mass [70]. These mucinous carcinomas are generally characterized by changes in MUC2 expression, both in terms of glycosylation and in levels of secretion. Patients with mucinous carcinomas generally exhibit elevated production of MUC2 in the GI tract; this may be correlated with low MUC2 gene methylation [71]. The elevated production and altered glycosylation of MUC2 has been correlated with increased metastatic and adhesion capability of these tumors [72], and siRNA therapies that decrease MUC2 expression have shown some anti-tumoral benefits [70].

Mucin underproduction can reduce the ability of the mucus layer to prevent pathogen diffusion to the epithelial cell layer, which can result in inflammation and infection [73]. In ulcerative colitis, an inflammatory bowel disease, pathogens adhere to the epithelial surface of the colon, which activates the immune response, causing small ulcers to form on the surface of the epithelium [74]. This disease can be caused by a reduction in the mucus-producing goblet cell population in the colon which reduces MUC2 production [75]. In addition, changes in MUC2 glycosylation, specifically, impaired production of core 1- and 3-derived *O*-glycans, have been observed in humans with ulcerative colitis [76].

In these disease states, certain cell surface proteins are upregulated and can provide potential opportunities to specifically target the diseased cells. In early adenocarcinomas, which causes mucin overproduction, the protein CEACAM6 has been shown to be overexpressed when compared to healthy surrounding tissue [77]. In addition, carcinoembryonic antigen (CEA) has also been indicated as a potential biomarker for colorectal cancers [78]. Upregulation of biomarkers such as peptide YY [79], alpha-1 antitrypsin [80], toll-like receptor 4 [81], and serum leucine-rich alpha-2 glycoprotein [82] within the ileum has been correlated with ulcerative colitis. Nano-drug delivery systems can take advantage of this increase in potential targets as well as changes in mucin production and glycosylation associated with these disease states. Antibodies that can recognize these targets can be used to localize nanoparticles to the specific disease sites, enabling more effective treatment.

### Mucus-interacting pathogens

Some types of bacteria, such as lactobacilli and enterococci, are able to adhere to mucus using mucus-binding moieties; other types, such as salmonella, are able to bind to the mucus layer using extracellular appendages [83].

Lactobacilli are a genus of bacteria that are important commensal members of the human GI tract [84]. Their main function is to convert sugars to lactic acid, though they have also been shown to inhibit the growth of harmful pathogens such as *H*.* pylori* and *C*.* albicans*. Lactobacilli use a number of mechanisms to adhere to mucus. Lactobacilli use proteins that promote mucus adhesion, such as mucus-binding proteins (MUBs) [84]. Examples of these MUBs include mucus adhesion-promoting protein (MapA) in *L*.* reuteri* [85] and Lam29 in *L*.* mucosae* [86]. These proteins extend out from the surface of the bacterial cell wall and contain a signal peptide that interacts with the carbohydrates found in the mucin glycoproteins. In addition, lactobacilli use multifunctional mucus adhesins found in the ATP-binding cassette transporter to bind to many types of GI surfaces, including GI mucus and collagen [87]. Enterococci are another genus of bacteria that can bind to the mucus in the GI tract; their mucus binding is promoted by the activity of the enzyme sortase A [88].

Other types of bacteria and pathogens that have been shown to adhere successfully to the mucus layers in the body include *E*.* coli* and salmonella, which have well-documented adverse effects on the GI tract [83]. These gram-negative bacteria adhere to the surface of the mucus layers using extracellular appendages such as flagella, fimbriae, and pili. Flagella are used primarily to improve bacterial motility in different environments, and their ability to adhere to the surface of the mucus layers is considered the first step in bacterial colonization of the mucus layer [83]. Flagella have been shown to aid the mucoadhesion of *E*.* coli* [89], salmonella [90], *C*.* difficile* [91], and *C*.* jejuni* [92], among other pathogens. Fimbriae are another type of bacterial extracellular appendage, which enable specific binding to different targets in the environment, as opposed to general adhesion with the flagella [83]. The fimbriae of the pathogens *E*.* coli* [93] and salmonella [94] contain adhesins that specifically bind to mucin glycans, which promotes adhesion to and aids colonization of the mucus layers, especially in the colon. Finally, pili are extracellular appendages that are similar to fimbriae; they are used by both gram-negative and gram-positive bacteria to improve adherence to surfaces in their environment [83]. While these have not been studied extensively, there is some evidence that pili improve adhesion to the mucus layer in gram-positive bacteria, both through specific binding [95] and electrostatic interactions [96].

Another class of mucus-interacting pathogens involves mucus-degrading pathogens (which can secrete mucolytic enzymes that compromise the integrity of the mucus layer). One classical example of a mucus-degrading pathogen is *A*.* muciniphila*, which is primarily found in the colon due to the favorable environmental parameters found there [97]. *A*.* muciniphila* degrades the mucus layer to obtain essential growth compounds such as l-threonine and GalNAc [97]. Another mucus-degrading pathogen is *B*.* thetaiotaomicron*, which degrades and utilizes *O*-glycans from mucins as a necessary step towards GI colonization [98]. A similar pathogen, *B*.* fragilis*, uses a mucin-desulfating sulfatase enzyme to degrade mucus in preparation for pathogenic colonization in the gut [99]. The protozoan *E*.* histolytica* secretes cysteine proteases that cleave MUC2-based mucus gels at the C-terminal domains, allowing for intestinal infection [100].

## Mucoadhesive drug delivery systems

Mucoadhesive materials, both naturally derived and synthetic, have been studied for decades, and a few mucoadhesive drug delivery systems have been used for FDA-approved therapies. This section will review the current understanding of mucoadhesion as well as the development of mucoadhesive drug delivery systems over time. Mucoadhesion has generally been shown to improve drug bioavailability [101–106].

Broadly, mucoadhesive drug delivery systems have longer gastric residence times, due to the interactions between the systems themselves and the components of GI mucus, as outlined above. These nanoscale or microscale drug delivery systems have been used primarily to deliver small molecules orally. While there are a large number of mucoadhesive polymers that have been investigated, we have chosen to focus on the most commonly investigated polymers—chitosan, carbomer, alginate, and cellulose—followed by a short discussion on more novel mucoadhesive polymer formulations. In these sections, we review the advances in developing these systems at the research-scale as well as their performance enhancement in **non-clinical animal models**; we will discuss commercial and clinical development of mucoadhesive systems, as well as other mucus-interacting systems, in a later section.

### Basis for mucoadhesion

**Fig. 3:**
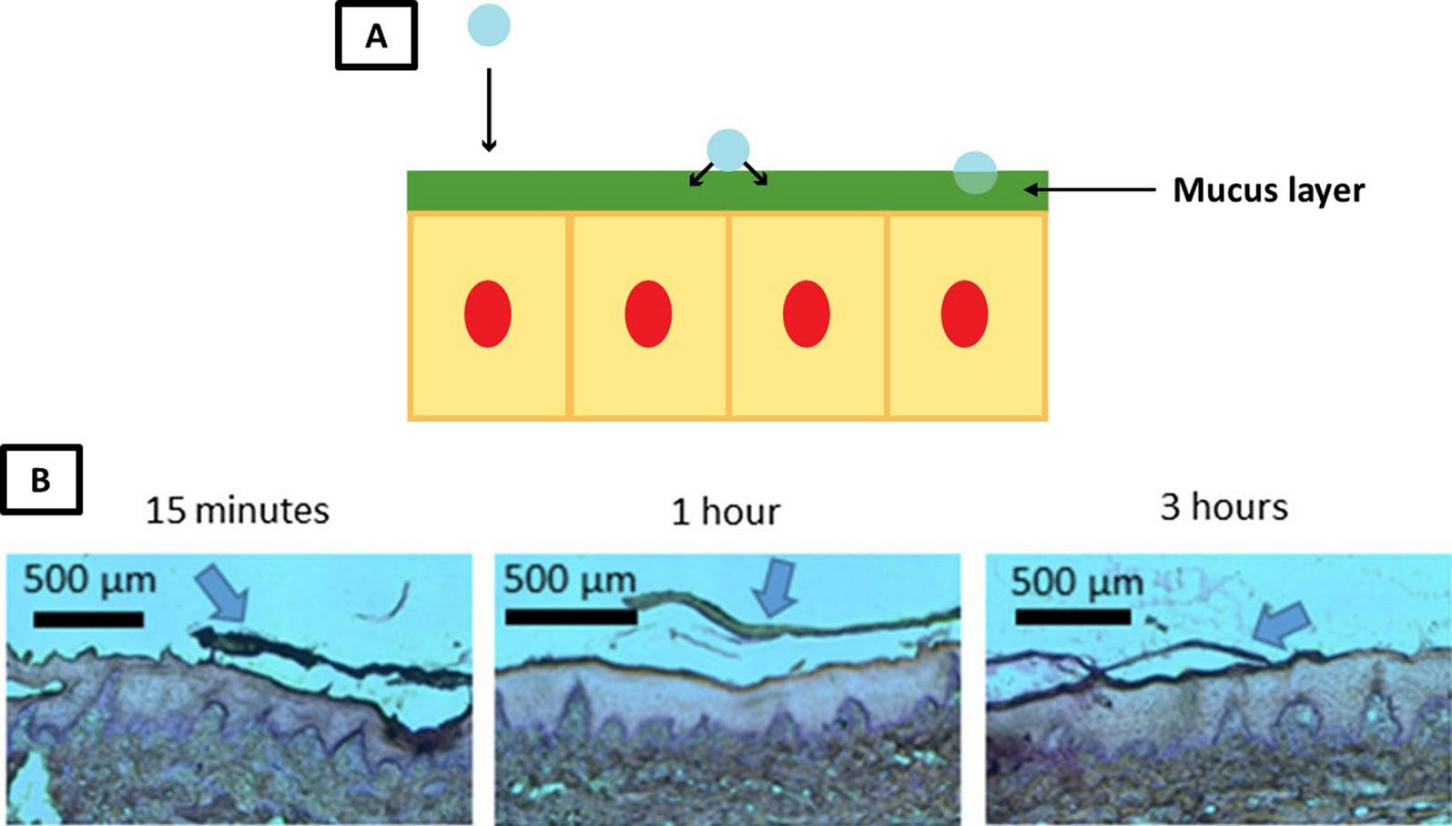
Schematic for the contact (left) and consolidation (middle) steps in forming a successful mucoadhesive bond (right) between a nanoparticle and the surface of the mucus layer (**A**), as well as a histological image of the mucoadhesion process for electrospun fibers (**B**) [119]. In this paper, the mucoadhesive bonds represent bonds with the mucus layers (composed of GI mucins) rather than with the GI mucosa (such as epithelial cells in the small and large intestines)

Mucoadhesion is a complex phenomenon, and many different types of materials will have interactions with mucus due to their large size and diverse composition [107]. The mechanism of mucoadhesion requires two main steps: contact and consolidation [107] (Fig. 3). First, the material must contact the mucus membrane in such a manner that the material cannot be dislodged by normal physiological actions in the surrounding area; i.e., the contact must be intimate. This is usually done in the GI tract through the normal movement of GI fluid, as the motion of the fluid will bring the material in contact with the GI mucus layers [107]. However, if the attraction between the material and the mucus layer is not strong enough to overcome repulsive forces that resist adhesion, such as the disruption caused by GI motion (for weaker adhesives) and natural mucus turnover (for stronger adhesives) [108], the particles would be displaced.

For prolonged adhesion, consolidation must take place; this involves a change in the properties of the mucus layer itself to strengthen the bond with the mucoadhesive material, so that the adhesion will hold in the face of potential dislodging stresses [107]. Consolidation has been explained with two different theories. The first, known as the dehydration theory, involves movement of water from the mucus layer to the mucoadhesive material until an equilibrium is reached [109]. This reduces the lubrication ability of the gel and promotes adhesion of the material [110], and is seen in polyelectrolyte gels, which have a high affinity for water and swell extensively upon exposure to water [111]. This was demonstrated through experimental observation of the flow of water from mucus gels to carbomer dry dosage forms by Mortazavi et al., as well as the resulting increase in detachment force and resistance to elastic deformation and viscous flow [110]. The second, known as the interpenetration theory, involves interpenetration of polymer chains across the material–mucus interface, which strengthens the bond between the material and the mucus. This theory was first proposed by Voyutskii [112] and explored on a theoretical basis by Peppas and Sahlin [113], in which improving the compatibility and similarity of the polymers with the mucus layers led to improved interpenetration and increased the mechanical strength of the material–mucus bond. Peppas and collaborators [114–118] further studied the interpenetration of polymer chains in mucoadhesive interactions and found that the magnitude of interpenetration was dependent on molecular characteristics such as polymer chain length and gel volume fraction.

There are a number of theories that explain how the attractive forces strengthen the material-mucus bond [107]. The electrostatic theory advocated by Derjaguin et al. [120] proposes that, upon contact, electrons are transferred between the two materials at the interface, leading to the formation of an electrical double layer at the interface and subsequent adhesion due to electrostatic effects. When the mucus layer adopts a liquid-like conformation, the wetting theory [113] says that the affinity of binding depends on the surface energies of the solid material and “liquid-like” mucus, along with the interfacial energy between the two materials. Materials which have a low interfacial energy with the mucus will cause the mucus to spontaneously “spread” across the material, increasing the number of material–mucus bonds. The theory of mechanical interlocking [113] proposes interlocking of the adhesive onto surface irregularities as the mechanism of adhesion; however, these interlocking forces have been shown to be less significant than the bond forces associated with the interpenetration theory described earlier. The general adsorption theory [113] proposes that the strength of mucoadhesion is dependent on the combined secondary attractive forces between molecules, i.e., van der Waals bonding and hydrogen bonding; these secondary forces have generally been accepted to be the primary contributor to the strength of adhesion, although the mechanism is probably similar to the contact and consolidation steps mentioned earlier.

Understanding the attractive forces and mechanisms of adhesion has allowed researchers to identify and develop materials that are particularly good mucoadhesives for use in prolonged drug delivery systems. The next sections will describe several materials that have been used in mucoadhesive drug delivery systems, as well as the history of their development and their current uses.

### Chitosan-based mucoadhesive systems

Chitosan has been widely studied as a mucoadhesive material, and is a biopolymer consisting of the monomer chitin—a glycosamine glycan [121]. Chitin is abundantly found in nature, particularly in the shells of aquatic life such as crabs and shellfish. Chitosan can be produced from chitin through deacetylation [122]. An important characteristic of chitosan-based systems is that they are cationic [121]; they are able to form electrostatic interactions with the negatively charged sialic groups present in the mucins, increasing the strength of the bond and providing greater resistance to dislodging forces. Indeed, the bioadhesive capability of chitin-based nanoparticles has been shown to be higher than control polymer nanoparticles; Bravo-Osuna et al. demonstrated a 10–50-fold increase in nanoparticle attachment when compared to control poly(isobutyl cyanoacrylate) nanoparticles, as measured through quantification of fluorescent nanoparticle adhesion to ex vivo segments of rat jejunum [123]. Higher bioadhesive capability can increase the gastric residence time, potentially by two to threefold [124], and makes the material an attractive candidate for prolonged drug delivery. Ling Tan et al. [125] used chitosan-coated nanoparticles to deliver amphotericin B and found an improvement in GI retention when compared to similar uncoated nanoparticles (63.9% and 56.1%, respectively). Imperiale et al. [126] used chitosan-based nanoparticles to deliver the protein drug interferon alpha, and found that the AUC (56 pg h/mL) and plasma concentration of the drug after 30 min (48.4 pg/mL) approximated that of subcutaneous injection. Murthy et al. [127] used self-assembled lecithin-chitosan nanoparticles to deliver raloxifene and found a ~ 4.2-fold increase in oral bioavailability when compared to a raloxifene suspension. Wang et al. [128] used chitosan nanoparticles to deliver metformin for the treatment of polycystic kidney disease and found a 1.3-fold increase in AUC when compared to free drug. Rosso et al. [129] used chitosan-based nanocomposite sponges to prolong residence time and found that the released drug was present for at least 6 h in the cecum (compared to 3 h for non-encapsulated drug). Shin et al. [130] investigated chitosan and oligochitosan-based coatings for curcumin nanosuspensions, and found a three to fourfold increase in mucoadhesion when compared to uncoated nanosuspensions. Cheng et al. [131] used chitosan-coated nanoparticles for insulin delivery and found that they exhibited a 16-fold increase in insulin AUC when compared to an oral solution, and roughly 80% of that of a subcutaneous injection of insulin. Abd El Hady et al. [132] synthesized chitosan–polyethylene oxide nanofibers for nizatidine delivery and found that nanofibers with an 8:2 ratio of chitosan to polyethylene oxide showed the highest mucoadhesive strength (22.82 g/cm^2^), as well as prolonged drug release when compared to control nanofibers.

Improvements in the mucoadhesion capability of chitosan-based materials have been made mainly by adding other biocompatible materials such as poly(ethylene glycol) (PEG) or changing the structural characteristics of the chitosan itself [133]. Two notable derivatives of chitosan are *N*-trimethyl chitosan chloride (TMC) and thiolated chitosan [134]. TMC is formed from chitosan through reductive methylation; this reaction can be controlled to obtain differing degrees of quaternization and produce different types of TMC polymers. These polymers show higher levels of mucoadhesion which correlate with higher degrees of quaternization [135]. This correlation may be due to the increase in positive charge density associated with quaternization, which could increase the strength of the electrostatic interactions. As an example, Ramalingam et al. [136] used TMC-based solid lipid nanoparticles to deliver resveratrol and found a 3.8-fold increase in oral bioavailability when compared to a resveratrol suspension.

Thiolated chitosan is formed when thiol-containing moieties are conjugated to chitosan. Four types of thiolated chitosan materials that have been synthesized for mucoadhesive development: chitosan-thioglycolic acid (Ch-TGA), chitosan-4-thiobutyl-amidine (Ch-TBA), chitosan-cysteine (Ch-Cys), and chitosan-thioethylamidine (Ch-TEA) [133]. The mucoadhesive properties of these thiolated chitosan materials are enhanced in two ways: electrostatic interactions increase mucoadhesion to the sialic acid regions, and disulfide bonds form with the cysteine-rich regions of the mucin proteins [137]. When compared to unthiolated chitosan, thiolated chitosan exhibited up to a twofold increase in mucoadhesion, as measured by the amount of attached nanoparticles [138, 139]. Millotti et al. [140] tested a variety of chitosan-6-mercaptonicotinic acid formulations and found an 80-fold increase in mucoadhesion and an improvement of up to 6.8-fold in AUC when compared to unthiolated chitosan. Similarly, Maria et al. [141] used preactivated thiolated chitosan nanoparticles to deliver octreotide, and found a 16-fold increase in mucoadhesion compared to unthiolated chitosan nanoparticles and a 7.2-fold increase in AUC when compared to free octreotide solution.

Commercially, chitosan has not been significantly incorporated into FDA-approved formulations when compared to other mucoadhesive materials—for more details, see Table 4.

### Carbomer mucoadhesive systems

Carbomer is the trade name for the polymer poly(acrylic acid) (PAA). There is a variety of different formulations of Carbomer available which vary in molecular weight and polymer architecture [142]. These polymers are biocompatible, and it is believed that they are not absorbed into the bloodstream during normal GI function due to their relatively large molecular weight; in addition, they exhibit mucoadhesive properties that make them attractive for localized and long-term oral drug delivery [142]. As such, these materials have been primarily used to improve bioadhesive properties for controlled drug delivery systems.

Carbomer-based materials were first synthesized and patented in 1957 [143]. Since then, research has focused on developing carbomer-based materials for oral drug delivery. The carboxyl groups in the monomer subunits in Carbomer are potentially able to form hydrogen bonds with the sialic acid and sulfate residues found on the oligosaccharide chains in the mucin proteins [144], making the polymer especially appealing for use in oral drug delivery [145]. Two major uses of carbomer materials in orally administered tablets are in the delivery of sodium fluoride and mesalamine. Bottenberg et al. described a method of preparing tablets composed of the carbomer 934P which contained sodium fluoride that were successfully delivered orally [146], while French and Mauger demonstrated successful preparation and oral administration of mesalamine-containing tablets composed of the carbomer 974P [147]. Sarkar et al. [148] developed carbomer-grafted gellan tablets for sustained release of metformin hydroxide and found that including the carbomer produced a large increase in retention time and a 30-fold increase in mucoadhesion strength when compared to ungrafted tablets. Compared to control (non-coated) liposomes, carbomer-coated liposomes exhibited up to a fourfold increase in adhesive capability in the intestine, according to Takeuchi et al. [149] Carbomer-coated liposomes exhibited up to a twofold increase in binding efficiency to pig mucin when compared to uncoated liposomes, according to the study conducted by Naderkhani et al. [150]. Ahmad et al. [151] showed that using carbomer-based microparticles for insulin delivery produced up to a 5.9-fold increase in insulin transport across the monolayer and increased oral bioavailability by up to 7.45 times when compared to an insulin solution.

As with other mucoadhesive materials, thiolation of carbomer-based systems has been shown to improve its mucoadhesion. Cevher et al. [152] demonstrated that among different carbomers (934P, 971P, 974P), the carbomer 934P-cysteine conjugate showed the highest work of mucoadhesion among all carbomers and thiolated conjugates and presented a twofold improvement over naïve carbomer 934P. Bonengel et al. [153] prepared thiol-modified alkylated carbomers and found a 9.2-fold improvement in mucus retention after 3 h when compared to unmodified carbomer.

Carbomer-based systems have been used commercially in FDA-approved systems, mainly as an inactive excipient that promotes mucoadhesion and long-lasting drug release; see Table 4 for more information about specific mucoadhesive systems that incorporate carbomer.

### Alginate mucoadhesive systems

Alginate is a naturally occurring polymer that is usually extracted from brown seaweed; it consists of alternating blocks of 1–4 linked α-l-guluronic acid and β-d-mannuronic acid residues [134]. Just as with Carbomer, these residues contain carboxyl groups that form hydrogen bonds with the sialic acid and sulfate residues found on the oligosaccharide chains present in mucins, thus creating a relatively strong bond with the mucus layers and enabling adhesion. In addition, the presence of the carboxyl groups increases the charge density, which increases the attractive forces between the material and mucus, enhancing adhesion. In contrast to chitosan, which contains a positive charge density, alginate is a polyanionic polymer in which the negatively charged compounds interact with the mucin layer to form hydrogen bonds, which increases the mucoadhesive strength [154]. There are many advantages to using alginate: it is more mucoadhesive compared to polycationic polymers and non-ionic polymers [155], and it is biodegradable (in contrast to PEG and carbomers, which are non-biodegradable) [134].

Gombotz et al. investigated the potential for mucoadhesion and protein release from alginate matrices [156], and showed that this material can be used for localization of oral drug delivery carriers. Long et al. [157] investigated the use of alginate-based nanoparticles for insulin delivery and found that these nanoparticles improved insulin activity by 25%, as measured by reduction in blood glucose level; in addition, they found that modifying the nanoparticles with vitamin B_12_ improved the permeation efficiency in the small intestine and improved insulin activity by 54%. Ghosal et al. [158] developed an interpenetrating network microbead consisting of alginate, poly(vinyl alcohol), and xanthan gum, and found that the microbeads exhibited strong mucoadhesion for over 6 h under neutral conditions. Azad et al. [159] used alginate microbeads to encapsulate peppermint oil and found that 94% and 36% of microbeads remained attached to the small intestine after 1 h and 6 h, respectively; they also showed an improved anti-inflammatory response when compared to control and loperamide treatments.

Thiolated alginate materials have also been synthesized through covalent linking of l-cysteine to alginate [160]. Just as with thiolated chitosan and carbomer, this modification increases mucoadhesion, enabling disulfide bonds to form with the cysteine residues in the mucin proteins [161]. Thiolated alginate has been shown by Bernkop-Schnürch et al. to increase the mucoadhesive ability of alginate by more than fourfold [162].

Alginate-based materials have not been commercially developed in FDA-approved systems to the same extent as other mucoadhesives; see Table 4 for more details.

### Cellulose-based mucoadhesive materials

Another category of mucoadhesive materials used for controlled oral drug delivery is cellulose-based materials. Cellulose is a linear chain of β(1–4) linked d-glucose units, and is primarily found in the cell walls of plants and some prokaryotic organisms. Cellulose derivatives such as methylcellulose (MC), ethylcellulose (EC), hydroxyethylcellulose (HEC), hydroxypropyl methylcellulose (HPMC), and carboxymethylcellulose (CMC) have been synthesized and investigated for mucoadhesive properties [163]. HEC and HPMC exhibit pH-dependent mucoadhesion to the different mucus layers; while HEC was mucoadhesive at a pH of 7 [164], HPMC exhibited optimal mucoadhesion at a pH of 6 [165]. Since cellulose-based materials are polyanionic, this change in mucoadhesion efficiency could be due to changes in material conformation; at lower pH, the high concentration of H^+^ ions in solution affect the structure of the material through interactions with the negatively charged groups, and this can lead to improved hydrogen bonding with the mucin layers [166]. EC and MC-based nanoparticles have been investigated by Suwannateep et al. for the oral delivery of curcumin [167]. EC-based nanoparticles demonstrated increased mucoadhesion when compared to EC-MC hybrid materials; however, EC-MC hybrid materials displayed a faster release of the curcumin. Xiong et al. [168] showed that encapsulation of resveratrol in ovalbumin-CMC nanoparticles increased the bioaccessibility of resveratrol to 80%, demonstrating an improvement from native resveratrol. Gadalla et al. [169] used pectin-NaCMC microspheres to deliver progesterone to the colon, and found a 1.8-fold increase in AUC and 2.3-fold increase in mean residence time when compared to a free solution of progesterone. Kaur et al. [170] developed EC nanoparticles for amphotericin B delivery for antifungal applications, and demonstrated a 15-fold improvement in oral bioavailability when compared to a free solution of amphotericin B.

As with other materials such as chitosan, carbomer, and alginate, thiolation of cellulose derivative materials enhances their mucoadhesion due to the formation of disulfide bonds. Nair et al. [171] synthesized nanoparticles from blends of HPMC and poly(lactide-*co*-glycolide) (PLGA) for the delivery of sitagliptin and demonstrated that they showed 52% retention in the stomach over 4 h. In addition, the HPMC and PLGA-based nanoparticles increased the residence time of sitagliptin in the GI tract: depletion occurred in 12 h, as compared to 5 h for an orally administered suspension of sitagliptin.

Cellulose (and its derivatives) have been widely used in FDA-approved commercial mucoadhesive systems, possibly due to their wide variety of tunable chemical and physical properties; see Table 4 for more information about the specific systems that utilize cellulose-based materials.

Table 2 summarizes the most used mucoadhesive materials, along with their potential modifications.

**Table 2:** Commonly used mucoadhesive materials and modifications

Material	Proposed mechanism of action	Possible modifications
Chitosan	Electrostatic interactions with sialic acid groups	Quaternization (trimethyl chitosan), thiolation
Carbomer	Hydrogen bonding with sialic acid and sulfate groups	Thiolation
Alginate	Hydrogen bonding with sialic acid and sulfate groups	Thiolation
Cellulose	Hydrogen bonding	Thiolation, derivatives (MC, EC, HEC, HPMC, CMC)

### Novel mucoadhesive materials

Most work in mucoadhesive materials has focused on using well-studied biocompatible materials such as chitosan, carbomer, alginate, and cellulose. However, in recent times (the last 15–20 years) novel materials have been investigated for mucoadhesive properties.

Wood et al. [172] developed complexation hydrogels (PEG-grafted poly(methacrylic acid) microparticles) functionalized with wheat germ agglutinin (WGA) and demonstrated an up to twofold increase in mucoadhesive capacity when compared to non-functionalized hydrogels. Catron et al. [173] conjugated 3,4-dihydroxy-l-phenylalanine [or levodopa (DOPA)], a compound found in mussel adhesive plaques, onto PEG-based polymers to improve their mucoadhesion. The mucoadsorption of the PEG-DOPA polymers was up to 3 times higher than that of other common mucoadhesive materials such as chitosan, poly(acrylic acid), and Gantrez polymers. Cheng et al. [174] investigated the use of poly(*n*-butylcyanoacrylate) nanoparticles for insulin delivery and found that the particles demonstrated good mucoadhesion, with approximately 70% retention after 12 h. Compared with the oral uptake of an insulin solution, they found a sixfold increase in the pharmacological availability of insulin (6.96%) and a 15.5-fold increase in bioavailability (7.74%). Amin et al. [175] examined the use of mobile composition of matter (MCM)-41 mesoporous silica nanoparticles (MSNs) for oral drug delivery and found that surface functionalization with polymers such as chitosan or PEG exhibited up to a threefold increase in mucin binding. Laha et al. [176] used propyl Karaya gum to form nanogels for the delivery of the antihypertensive drug bosentan monohydrate and found that these nanogels had a mucoadhesion of 42.69% after 8 h, demonstrating their mucoadhesive abilities. Cheng et al. [177] developed keratin-based nanoparticles for the delivery of amoxicillin and found that controlling the weight ratio of keratin to keratose could result in up to 80% gastric retention after 8 h and up to a 1.4-fold increase in AUC when compared to a pure amoxycillin oral dose. Harloff-Helleberg et al. [178] explored the mucoadhesive behavior of sucrose acetate isobutyrate (SAIB) and found an 11-fold increase in intestinal residence time when compared to a free solution. Zhao et al. [179] developed a nanoparticle self-assembled bioadhesive coacervate coating for inflammatory bowel disease treatment and demonstrated a retention time of more than 2 days and improved efficacy (as shown by a four to sixfold improvement in colonic histopathology score) when compared to an untreated control and oral administration of a solution of the drug.

Other mucoadhesive strategies have focused on mimicking the structural characteristics of mucus-binding pathogens (as described earlier). Walker et al. [180] took inspiration from the activity of *H*.* pylori* flagella to design micropropeller-based drug delivery systems that can penetrate mucin gels. A similar approach was employed by Choi et al. [181], where urease-powered polydopamine “micromotors” mimic the behavior of *H*.* pylori* to prolong retention in the stomach. Yang et al. [182] developed germ-mimetic nanoparticles that used different types of PEG chains to mimic the actions of flagella, using tip-specific extended PEG, and fimbriae, using packed PEG chains on the body, ultimately resulting in up to an 83-fold increase in nanoparticle diffusion and 21.9-fold increase in oral bioavailability of chemotherapeutic drugs. Wang et al. [183] developed chiral mesoporous silica nano-screws that mimic the action of helical bacteria for improved mucoadhesion, retention, and drug release (up to 5.65-fold improvement in AUC) when compared to mesoporous silica nanoparticles and nano-rods. Tang et al. [184] mimicked the ectocellular structure of *C*.* neoformans* to design nanoparticles that could effectively bind to the mucus layers for antimicrobial applications. Cai et al. [185] developed adhesive microparticles for dexamethasone that mimic the adhesive behavior of Boston ivy tendrils; they found a tenfold increase in adhesive performance in vivo when compared to similarly composed spherical particles, as well as improved performance against ulcerative colitis as shown by the decreased colon/body weight ratio when compared to control and administration of dexamethasone solution. Chen et al. [186] developed a microneedle delivery system that mimics the thorny-headed intestinal worm, combining physical and chemical methods of adhesion to improve the oral delivery and intestinal absorption of semaglutide.

## Passive mucus-penetrating systems: diffusion through mucus

The other major class of mucus-interacting systems are mucus-penetrating systems. In contrast to mucoadhesive systems, mucus-penetrating materials attempt to move through the mucus layer (rather than adhering to the surface of the mucus layer) and attach to the epithelial surface layer. This could give them potential advantages in delivering larger and more environmentally sensitive drugs such as peptides, due to their ability to release at the epithelium itself rather than within the lumen.

### Basis for mucus penetration

Passive mucus penetration occurs when nanoparticles diffuse through the mucus layer. As described earlier, mucus layers prevent or retard particle diffusion towards the epithelial surface. Consequently, most nanoparticles do not penetrate the mucus layer effectively due to interactions and entanglements between the nanoparticle and the mucus. This presents a major challenge in oral drug delivery, since it is often necessary to deliver drug cargo to the bloodstream. Mucus-penetrating systems could more efficiently deliver drug to the site of absorption by overcoming the entrapment of the nanoparticles or drugs within the mucus [187].

Passive mucus-penetrating properties arise by minimizing the interactions between the nanoparticle surface and the mucus layers [163]. Entanglement is the biggest obstacle for nanoparticle penetration; reducing entanglement would enable nanoparticles to move through the mucus layer. A significant contributor to nanoparticle-mucus interactions arises from hydrogen bonding and electrostatic interactions with the charged sialic acid groups in the mucus constituents; therefore, reducing the net charge density would diminish these interactions and promote nanoparticle diffusion. To reduce the charge density on the surface of the nanoparticle, it can be covered with either an uncharged biocompatible material or a highly dense, evenly distributed assortment of an equal amount of positive and negative charge [187].

### Low-molecular weight PEG coatings

One method of designing a passive mucus-penetrating system is by coating nanoparticles with low-molecular weight PEG. PEG is a hydrophilic and biocompatible polymer widely used in biomedical applications. PEG minimizes interactions between nanoparticles and mucus, enabling easier penetration [188]. Its neutral charge makes it ideal for minimizing interactions with mucins. The most important factor in improving mucus penetration with PEG was the ability to densely coat the surface of the nanoparticle; this was most easily done using low-molecular weight PEG_5000_ [189]. Increasing the molecular weight of PEG (for example, using PEG_10000_) generally increased entanglement with the mucin chains, which decreased mobility of the nanoparticles through the mucus layers [190]. Some groups posit that there is an optimal PEG molecular weight for mucus penetration; Mert et al. found that PEG_1000_-coated PLGA nanoparticles had a 33-fold lower mean square displacement within human cervicovaginal mucus when compared to PEG_5000_-coated PLGA nanoparticles [191]. However, groups have been able to develop methods of densely packing higher molecular weight PEG (ranging from 10 to 40 kDa) onto the surface of nanoparticles, and these have shown improvements in mucus penetration as well [192–194]; this demonstrates that the most important criterion is the ability to densely coat the PEG onto the surface rather than the molecular weight of the PEG itself.

Anderski et al. [195] investigated the use of PEG coatings for mucus-penetrating PLGA nanoparticles designed to deliver photosensitizers for photodynamic treatment of intestinal cancer; they found that PEG-coated nanoparticles had a 1.9- and 2-fold increase in penetration depth when compared to unmodified nanoparticles and chitosan-coated nanoparticles, respectively. Tan et al. [196] developed electroneutral mesoporous silica nanoparticles with poly(lactic acid) (PLA)-PEG and cell-penetrating peptide (CPP) modification, and found a decrease of up to ~ 50% in mucus trapping when compared to unmodified mesoporous silica nanoparticles. Guo et al. [197] investigated a number of different nanoparticle properties and their effects on mucus penetration and found that PLGA-PEG-PLGA nanoparticles exhibited 1.58-fold improvement in mucus penetration when compared to PLGA nanoparticles; in addition, incorporating PEG_2000_ within the triblock copolymer showed improved mucus penetration when compared to PEG_1000_ and PEG_500_. Sato et al. [198] investigated the use of polystyrene-PEG diblock copolymer nanoparticles for cyclosporine A delivery and found a 50- and 2-fold increase in bioavailability when compared to crude cyclosporine A and polystyrene-polyacrylic acid copolymer nanoparticles, respectively. Warren et al. [199] tested the effect of coating bovine milk exosomes with PEG_2000_ for siRNA delivery, and found that coating with PEG_2000_ improved the permeability coefficient in mucin threefold when compared to uncoated milk exosomes. Le et al. [200] developed lipid-polymer hybrid nanoparticles with a PEG coating for inflammatory bowel disease treatment and found a three–fourfold improvement in mucus penetration of PEGylated nanoparticles when compared to free superoxide dismutase in solution. Goto et al. [201] investigated PEGylated poly(methacrylic acid) microparticles and found that 70% of microparticles remained attached to the duodenum, as compared to 56% of non-modified poly(methacrylic acid) microparticles and 43% of control polystyrene microparticles. Puranik et al. [202] developed PEGylated polyanionic formulations for pH-responsive drug delivery and found substantial mucoadhesion at a concentration of 0.5 mg/mL based on energy dissipation measurements.

Tang et al. found that formulating nanoparticles out of a diblock poly(sebacic acid) (PSA)-PEG copolymer resulted in only 12-fold diffusion retardation (as measured by the calculated effective diffusion coefficient) in cervicovaginal mucus when compared to water, as opposed to a ~ 3300-fold diffusion retardation in cervicovaginal mucus for nanoparticles composed of PSA or poly(lactic-*co*-glycolic acid) (PLGA) [203]; this indicates that incorporating PEG on the surface of a nanoparticle formulation (which occurred with this diblock copolymer) has a significant benefit for mucus penetration. Lai et al. demonstrated the potential use of PEGylation to improve mucus penetration of larger nanoparticles (~ 200–500 nm). In their experiments, they found that modifying 200 nm polystyrene (PS) nanoparticles with PEG attachment resulted in 400-fold improvement in mean square displacement and 380-fold improvement in effective diffusion coefficient when compared to COOH-modified PS nanoparticles, while PEG attachment to 500 nm PS nanoparticles resulted in ~ 1100-fold improvement in mean square displacement and effective diffusion coefficient when compared to COOH-modified PS nanoparticles [188]. Their experiments also demonstrated that PEGylation significantly reduced the fraction of immobile nanoparticles, particularly for the 200 nm and 500 nm nanoparticles.

### Poloxamer-based mucus-penetrating systems

Another widely studied class of polymers used to coat nanoparticle surfaces in order to promote passive mucus penetration are poloxamers. Poloxamers are co-polymers containing PEG and poly(propylene glycol) (PPG) subunits; since the PEG subunits are hydrophilic and the PPG subunits are hydrophobic, the overall polymer is amphiphilic and thus does not promote hydrogen bonding with the mucus components [204]. In addition, poloxamers are non-ionic and thus reduce electrostatic interactions with the mucins [163]. The most commonly used poloxamer for mucus penetration is the Pluronic class of poloxamers [187]; Pluronics are triblock PEG–PPG–PEG copolymers. These poloxamers can be tuned by adjusting the molecular weight of the copolymer and the PPG/PEG ratio, which affects their transport properties. Pluronic polymers were first studied in the 1950s [205] and have been used in a number of drug delivery devices due to their extensive safety profile.

Pluronic is an FDA-approved material for mucus-penetrating systems and has been used to enhance the penetration of different types of drug delivery systems into mucus layers. Yang et al. [187] investigated how changing the molecular weight of the Pluronic coatings affected the penetration of fluorescently tagged PLGA nanoparticles through human cervical mucus. They found that increasing the molecular weight of the Pluronic coating improved the ability of the nanoparticle to move through the human cervical mucus. In particular, coating the PLGA nanoparticles with the coating Pluronic F-127 significantly improved the penetration of the nanoparticles through the human cervical mucus, with a 280-fold increase in the mean squared displacement of the particles and over an 80-fold increase in penetrable fraction when compared to uncoated PLGA nanoparticles. In addition, Li et al. [206] demonstrated that liposomes coated with the same Pluronic F-127 coating exhibited a five to sevenfold increase in diffusion efficiency when compared to uncoated liposomes. Chen et al. [207] showed that coating the surfaces of liposomes with Pluronic F-127 improved the concentration of cyclosporine A in plasma over long periods of time when compared to unmodified liposomes and chitosan-coated liposomes, with a 1.5 to 2-fold increase in drug transportation to the underlying tissue and a 1.25 to 2-fold increase in AUC. Fares et al. [208] used a mixture of the Pluronics P123 and F-127 to create polymeric micelles which encapsulated lacidipine; they demonstrated that using the micelles resulted in a 6.85-fold increase in lacidipine bioavailability when compared to a lacidipine suspension. Huang et al. [209] functionalized PLGA nanoparticles with Pluronic F-127 for curcumin delivery and found a ~ 10% increase in migration distance of the nanoparticles through mucus when compared to non-functionalized nanoparticles. Date et al. [210] formulated a budesonide nanosuspension coated with Pluronic F-127 and found superior treatment of inflammatory bowel disease when compared to non-treatment and treatment with a polyvinylpyrollidine-coated budesonide microsuspension. Song et al. [211] used Pluronic F-127 as a shielding agent for delivery of cyclosporine A via self-nanoemulsifying systems and found that including Pluronic F-127 increased the drug bioavailability by up to twofold and the cellular uptake by up to 3.5-fold when compared to non-modified self-nanoemulsifying systems.

### Virus-mimicking drug delivery systems

The design of virus-mimicking nanoparticles is based on the successful mucus penetration of viruses such as capsid viruses, which readily infect mucosal surfaces, and closely related viruses such as Norwalk and human papilloma viruses; this was first demonstrated by Olmsted et al. in cervical mucus [33, 42]. In some cases, these viruses can diffuse through mucus almost as quickly as they can diffuse through aqueous or saline solutions [42]. The major structural characteristic of these viruses that enables them to penetrate mucus is their high-density surface charge coating, which contains equal densities of positive and negative charges much like that of soluble proteins [212]. Bond formation between the virus’s surface and the mucin chains is reduced in two ways. First, the high density of the charge coverage reduces the exposure of hydrophobic domains on the surface of the virus, which could form nonpolar interactions with the mucin chains [33]. Secondly, the net neutral charge on the virus reduces the electrostatic interactions formed with the negatively charged sialic acid groups in the mucin proteins [213]. Nanoparticles can be designed to display the same surface charge characteristics—dense coverage and a net neutral charge—that are found in these viruses.

Pereira de Sousa et al. [214] designed nanoparticles with highly densely charged surfaces, which contained a combination of cationic chitosan and anionic chondroitin sulfate. They found that these virus-mimicking nanoparticles exhibited a threefold increase in diffusion ability within porcine intestinal mucus when compared to unmodified nanoparticles. Pereira de Sousa et al. [215] also combined the virus-mimicking strategy with the PEG shielding strategy described in the previous section and found that the virus-mimicking plus PEG nanoparticles exhibited a fivefold increase in mucus penetration when compared to unmodified nanoparticles. Wu et al. [216] used a combination of cationic octa-arginine peptide and anionic phosphoserine to create virus-mimicking nanoparticles which increased the bioavailability of insulin by 1.9-fold compared to non-virus-mimicking nanoparticles; their nanoparticle also exhibited a similar mucus penetration speed when compared to PEG-shielded nanoparticles. Bao et al. [217] designed virus-mimicking self-assembled α-lactalbumin peptosomes for curcumin delivery and found that one specific formulation (short nanotubes) had a retention time of 8 h in the small intestine and a 6.85-fold increase in AUC when compared to free curcumin. Cheng et al. [218] synthesized folic acid-coated virus-mimicking poly(*n*-butylcyanoacrylate) nanoparticles for oral insulin delivery and found that grafting folic acid at a ratio of at least 12.51% resulted in superior mucus penetration in the duodenum, jejunum, and ileum, as well as similar AUC when compared to a subcutaneous injection of insulin. Zhang et al. [219] coated mesoporous silica nanoparticles with both a cationic cell-penetrating peptide and an anionic glutaric anhydride to create a virus-mimicking nanoparticle that exhibited a 2.1-fold improvement in insulin bioavailability when compared with directly administered insulin in the jejunum.

Han et al. [220] used a similar concept (zwitterionic nanoparticles and micelles) to improve mucus penetration for oral insulin delivery; the zwitterionic particles showed a 6.7- and ~ 100-fold improvement in mean squared displacement (MSD) through porcine mucus when compared to PEG-conjugated nanoparticles and anionic/cationic nanoparticles, respectively, while zwitterionic micelles showed a 12-fold improvement in MSD when compared to PEG-covered Polysorbate 80 micelles. Similarly, Gao et al. [221] developed zwitterion-functionalized mesoporous silica nanoparticles for oral delivery of protein drugs and found a ~ 1.33-fold improvement in mucus penetration in vitro and up to ~ fourfold improvement in rat intestine permeation when compared to non-functionalized nanoparticles. Rao et al. [222] coated porous silicon nanoparticles with polyphosphoester and the zwitterion dodecyl sulfobetaine for insulin delivery, and found a twofold increase in the permeability coefficient in mucin compared to non-zwitterionic nanoparticles and a twofold increase in insulin oral bioavailability when compared to free insulin solution. Biosca et al. [223] developed zwitterionic self-assembled nanoparticles for targeting Plasmodium strains to improve antimalarial bioavailability and found a ninefold increase in blood drug concentration when compared to free solution administration. Hu et al. [224] developed zwitterionic polydopamine-modified PLGA nanoparticles and showed that they demonstrated superior mucus penetration (by at least tenfold improvement in mean particle displacement) when compared to unmodified PLGA nanoparticles.

## Active mucus-penetrating systems: mucolysis

Active mucus-penetrating systems, also known as mucolytic systems, have not been studied as extensively as passive mucus-penetrating systems, as they generally result in disruption of the mucus layer itself. While mucus layer disruption can increase the diffusion of drugs or drug-containing nanoparticles to the epithelial surface, it also increases the risk of pathogen diffusion and subsequent infection of the epithelial cells. However, mucolytic materials have been studied for some time and have been used in GI therapies to reduce the thickness of the mucus layer.

Disulfide-breaking agents have been used as mucolytics to improve the efficacy of delivery to the epithelia; one in particular is *N*-acetylcysteine (NAC). NAC is a part of a class of materials known as sulfhydryl compounds [225]; sulfhydryl compounds contain a free sulfur group that can readily form disulfide bonds with cysteine groups in the mucin subunits. The disulfide bonds between the mucin chains are cleaved, ultimately reducing the cross-linking present in mucus gels, enabling nanoparticles to penetrate the mucus layer [225]. The major downside to using a general disulfide-breaking agent, however, is the potential for wide-scale mucus cleavage, which risks exposing the epithelium to pathogens and other foreign materials [163]. To address this issue, the disulfide-breaking agents are generally incorporated into the nanoparticles. This enables gradual release of the breaking agent from the nanoparticle over time [226] and allowing only localized mucus clearance. NAC was first studied as a mucolytic by Sheffner in 1963 [227] and has been used as a mucolytic for certain respiratory diseases. Recently, it has been investigated as a potential permeation enhancer: Takatsuka et al. [228] showed that administering NAC along with a drug formulation increased its bioavailability by threefold when compared to administering just the drug formulation. Tian et al. [229] encapsulated a PEG–NAC conjugate within a nanostructured lipid carrier to deliver curcumin; they found that perfusion was increased up to threefold throughout the intestine and AUC increased up to 500- and 117-fold when compared to curcumin solution and unmodified curcumin nanostructured lipid carriers, respectively. Similar conjugates such as thiobutylamidine-dodecylamine and thioglycolic acid-octylamine were investigated by Rohrer et al. [226] and found similar mucolytic capabilities as solutions of the known mucolytics *N*-acetylcysteine and dithiothreitol.

Other approaches have immobilized mucolytic enzymes onto the surface of nanoparticles, allowing the enzymes to interact with the mucus layers only when the nanoparticle is proximal to mucus. The approach is useful because it localizes the cleavage of the mucus layer to the area in which the nanoparticles are diffusing, reducing the impact on the integrity of the mucus layer as a whole. Enzymes that have been used for this purpose include papain, bromelain, and trypsin [230]. There has been some work done on investigating the properties of enzyme-mediated mucolysis for oral drug delivery. Müller et al. [231] investigated the effect of conjugating papain to the surface of poly(acrylic acid)-based nanoparticles, and found that papain increased the penetration capability by 2.5-fold in vitro. Samaridou et al. [230] tested the effect of conjugating trypsin, papain, and bromelain to the surface of PLGA nanoparticles; compared to undecorated PLGA nanoparticles, trypsin-decorated nanoparticles enabled a twofold increase in permeability within porcine intestinal mucus, while papain-decorated and bromelain-decorated nanoparticles enabled a threefold increase in permeability. Pereira de Sousa et al. [232] utilized both papain and bromelain as mucolytic enzymes on nanoparticles and found that bromelain-decorated nanoparticles enabled a 4.8-fold increase in permeability compared to papain-decorated nanoparticles. Zafar et al. [233] decorated polycarbophil nanoparticles with papain for amoxicillin delivery, and found a ~ fivefold increase in deep penetration (34 mm) of GI mucus ex vivo when compared to non-decorated nanoparticles. Efiana et al. [234] modified self-emulsifying drug delivery systems (SEDDS) with 0.5% papain-palmitate and found a threefold increase in mucus permeability when compared to unmodified SEDDS. Razzaq et al. [235] synthesized papain-functionalized thiolated redox multifunctional polymeric micelles for delivery of paclitaxel to solid tumors and demonstrated a 7.89-fold improvement in mucus penetration when compared to pure paclitaxel.

One area that should be investigated further with this approach is keeping the immobilized mucolytic enzymes stable and protecting them from degradation in the stomach, since proteolytic enzymes such as pepsin can potentially denature the exposed enzymes and thus render them unable to successfully cleave the mucin layers. Recently, Homayun et al. [236] developed a co-delivery microparticle system, in which a lactase-loaded microparticle contained embedded halloysite nanotubes containing bromelain for mucus cleavage; they found that utilizing this system allowed for either partial or complete disruption of the mucus layer (and thus improved absorption efficiency), depending on the amount of bromelain loaded into the nanotubes.

Table 3 summarizes the materials used for mucus-penetrating and mucolytic systems, along with their proposed mechanism of action.

**Table 3:** Commonly used methods of developing mucus-penetrating systems

Material/characteristic	Type of system	Proposed mechanism of action
PEG	Mucus-penetrating	Reduced electrostatic interactions from densely packed neutrally charged surface
Poloxamer/Pluronic	Mucus-penetrating	Reduced electrostatic interactions from densely packed neutrally charged surface
Virus-mimicking	Mucus-penetrating	Reduced exposure of hydrophobic groups, reduced electrostatic interactions from net neutrally charged surface
Zwitterionic	Mucus-penetrating	Reduced exposure of hydrophobic groups, reduced electrostatic interactions from net neutrally charged surface
Mucolytic enzyme release	Mucolytic	Disulfide bond cleavage within the area of enzyme release
Mucolytic enzyme surface conjugation	Mucolytic	Disulfide bond cleavage at the site of nanoparticle-mucus interaction

**Fig. 4:**
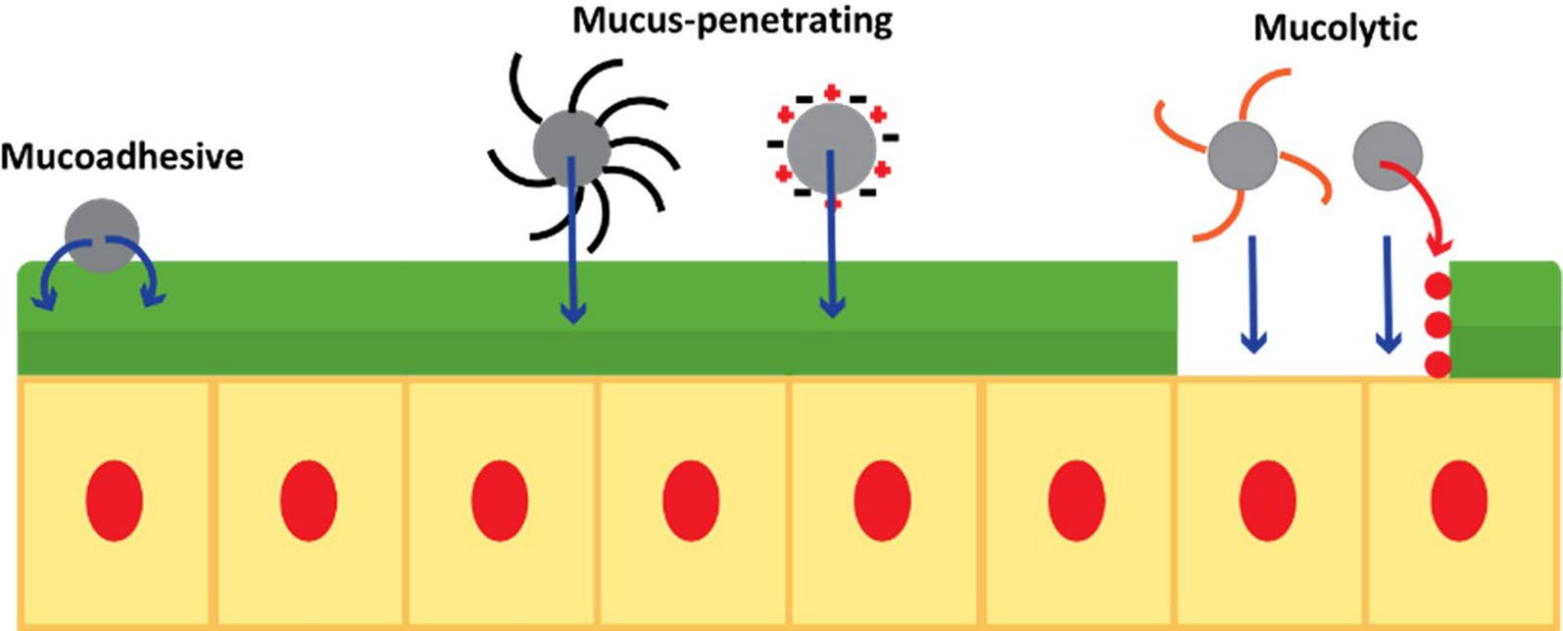
Illustration of the mucus-interacting methods employed for successful oral drug delivery: mucoadhesive, mucus-penetrating (densely layered uncharged surface coating and evenly distributed positive/negative surface charges) and mucolytic (conjugated and released mucolytic enzymes)

A complete schematic of mucus-interacting systems is shown in Fig. 4.

## FDA-approved mucus-interacting systems

Table 4 shows a list of some FDA-approved mucus-interacting drugs and drug formulations for various diseases, as well as excipients that have been included in other formulations that have mucus-interacting properties.

**Table 4:** FDA-approved mucus-interacting systems and excipients

Drug/formulation	Year of FDA approval	Type of mucus-interacting system	Function/purpose	References
MuGard	2006	Mucoadhesive (Carbomer)	Treatment of mucositis	[237]
Sitavig (acyclovir)	2013	Mucoadhesive (cellulose)	Buccal cold sore treatment	[238]
Oravig	2010	Mucoadhesive (cellulose)	Buccal mouth/throat yeast infection treatment	[239]
ProctiGard	2014	Mucoadhesive (Carbomer)	Treatment of rectal mucositis	[240]
SP1049C	2008 (Orphan Drug designation)	Mucus-penetrating (Pluronic)	Pluronic-based treatment of gastric carcinomas	[241]
Cetylev (*N*-acetylcysteine)	2016	Mucolytic (*N*-acetylcysteine conjugation)	Acetaminophen overdose treatment	[242]
Diphenoxylate hydrochloride and atropine sulfate	1978	Mucoadhesive (cellulose)	Diarrhea treatment	[243]
Tarka (trandolapril and verapamil hydrochloride extended release)	1996	Mucoadhesive (cellulose)	High blood pressure treatment	[244]
Kadian (morphine sulfate extended release)	1996	Mucoadhesive (Ethylcellulose)	Long-term severe pain treatment	[245]
Uroxatral (alfuzosin hydrochloride extended release)	2003	Mucoadhesive (Ethylcellulose, methylcellulose)	Benign prostatic hyperplasia treatment	[246]
K-Tab (potassium chloride extended release)	1980	Mucoadhesive (cellulose)	Hypokalemia treatment	[247]
Exalgo (hydromorphone hydrochloride extended release)	2010	Mucoadhesive (Cellulose acetate)	Management of moderate/severe pain in opioid-tolerant patients	[248]
Lescol XL (fluvastatin sodium extended release)	2000	Mucoadhesive (Hydroxypropylcellulose)	High cholesterol treatment	[249]
Mirapex (pramipexole dihydrochloride extended release)	1997	Mucoadhesive (Carbomer)	Parkinson’s disease treatment	[250]
Voltaren-XR (diclofenac sodium extended release)	1996	Mucoadhesive (Hydroxypropyl methylcellulose)	Osteoarthritis and rheumatoid arthritis symptom treatment	[251]
Kapspargo Sprinkle (metoprolol succinate extended release)	2018	Mucoadhesive (Ethylcellulose)	Angina, heart failure, high blood pressure treatment	[252]
Glumetza (metformin hydrochloride extended release)	2005	Mucoadhesive (cellulose)	Type 2 diabetes treatment	[253]
Razadyne ER (galantamine hydrobromide)	2005	Mucoadhesive (Ethylcellulose)	Alzheimer’s disease treatment	[254]
Trokendi XR (topiramate)	2013	Mucoadhesive (Ethylcellulose)	Epilepsy treatment	[255]
Wellbutrin XL (bupropion hydrochloride)	2003	Mucoadhesive (Ethylcellulose)	Major depressive disorder treatment	[256]
Elepsia XR (levetiracetam)	2018	Mucoadhesive (Ethylcellulose)	Partial-onset seizure adjunctive therapy	[257]
Aciphex (rabeprazole sodium delayed release)	1999	Mucoadhesive (Ethylcellulose)	Gastroesophageal reflux disease and duodenal ulcer treatment	[258]

Excipient	Year of FDA approval	Type of mucus-interacting system	Function/purpose	References
Carbopol 971P	2012	Mucoadhesive	Inactive ingredient in extended release tablets	[259]
Poloxamer 407	2016	Mucus-penetrating	Inactive ingredient in opioid-induced constipation treatment tablets (RELISTOR)	[260]
Poloxamer 188	1995, 2000	Mucus-penetrating	Inactive ingredient in antiprotozoal suspension (MEPROM) and antimalarial suspension (MALARONE)	[261, 262]

As can be seen from the table, there are more mucoadhesive treatments and excipients than mucus-penetrating and mucolytic systems that have been approved by the FDA, based on our review of the FDA-approved drug database. From our review of the literature, mucoadhesives have been more widely studied and do not have the same safety risks as mucus-penetrating and mucolytic systems. However, as more mucus-penetrating and mucolytic systems are studied and evaluated by the FDA, it is likely that more of these systems will receive FDA approval.

## Conclusions and future directions

Oral drug administration is the preferred route of drug delivery due to the ease of administration, which results in greater patient compliance. A major obstacle to oral drug delivery is the presence of the mucin layer covering the surface of the GI tract. While this mucin layer plays a critical role in protecting the epithelial surface from pathogens and harmful foreign substances, it also impedes the movement of drugs and drug carriers towards the epithelial surface, which reduces the bioavailability of orally delivered drugs. Understanding the composition and function of mucus and the current methods of interacting with the mucus layers for oral drug delivery treatments is necessary to improve the residence time of these treatments.

The mucus layer is a complex arrangement of mucin glycoproteins which has specific compositions and pore sizes that allow the mucus to perform their function. The mucus layers are cleared periodically; any entrapped material is removed for excretion. Mucus layers can be altered during disease; some microorganisms are able to adhere to mucus to prevent rapid clearance. These same strategies can be utilized to design drug carriers that can adhere to mucin and resist rapid excretion in order to improve oral drug pharmacokinetics. One area for further study is differential targeting, which takes advantage of variations in glycobiology that result from different disease states. Specifically targeting diseased mucus layers improves localization of orally administered therapies, minimizing the amount of drug that acts on healthy mucus, which both improves the treatment efficacy and reduces the potential for harmful side effects.

The three major classes of systems that have been used to improve interactions with the mucin are mucoadhesive, passive mucus-penetrating, and active mucus-penetrating (mucolytic) systems. Mucoadhesive systems are the most widely studied; these systems increase the interaction between the nanocarrier and the mucin surface through interpenetration and via secondary bonds. Nanoparticles that “stick” to the surface of the mucin resist the normal actions of GI clearance. Some polymers that have been investigated for their mucoadhesive properties are chitosan, carbomers, alginate, and cellulose-based polymers. These mucoadhesive materials have been used for the nanoparticles themselves and as coatings to improve mucoadhesion of currently existing polymer nanoparticles. Thiolation also improves mucoadhesion by increasing the potential for disulfide bonds to form between the material and mucin.

By contrast, passive mucus penetration and mucolytic systems attempt to minimize or control interactions with mucin, in order to avoid entanglement and enable greater penetration through the mucus layer. In passive mucus penetration systems, interactions between the surface of the nanoparticle and the mucin layers are minimized, which involves coating the surface with a material that both has a dense charge density and a net negative charge. Coating materials include poloxamers such as Pluronic F-127, low molecular weight PEG, and chitosan/chondroitin sulfate. More recently, ionic liquids have been used to reduce mucus viscosity and enabling greater mucus penetration by encapsulated drugs.

In mucolytic systems, the mucus layer is cleaved in a controlled manner by exposing it to mucolytic substances. There are two types of mucolytic systems: In one, disulfide breaking agents such as *N*-acetylcysteine are slowly released during particle diffusion, so as not to disrupt the integrity of the entire mucus layer. In another, mucolytic enzymes such as papain, bromelain, and trypsin are immobilized to the surface of the nanoparticle. Compared to passive mucus penetration methods, these methods are much more efficient at reducing mucin viscosity, enabling quick transport of drugs and drug nanocarriers to the epithelial surface; however, these methods are not as widely applied due to concerns about the integrity of the mucus layer and risk of infection.

When comparing mucoadhesive systems with passive and active mucus-penetrating systems, is it clear that they present two very different paradigms for prolonging GI residence. Mucoadhesive systems seek to “anchor” the system at the site of mucus and rely on their strong interactions with the GI mucus to prolong residence; as such, they are attractive candidates for long-term drug delivery (though their residence is limited by mucus turnover, as discussed earlier). However, one major drawback of mucoadhesive systems is that these systems cannot access the underlying epithelium (due to their strong interactions with the mucus); this could influence their possible applications if the drug of interest is large enough, since these drugs will have difficulty penetrating the mucus layer. Mucus-penetrating systems, on the other hand, bypass the mucus layer (by design) and interact directly with the epithelium. These systems, while not as widely studied as mucoadhesive systems, have greater potential for ultra-long term drug delivery (because their clearance relies on epithelial cell turnover, which has been approximated as occurring every 3–5 days [263] as opposed to the much shorter timeframe (1–7 h) of GI mucus turnover [21, 63, 64]). In addition, they may provide superior protection for sensitive drug cargo such as peptides, due to reduced exposure to the digestive enzymes in the GI lumen, and they may result in improved bioavailability for larger drugs because the released drugs would not have to penetrate the mucus barrier prior to uptake in the epithelium. However, using these systems (particularly mucolytic systems) may result in temporary or longer-term damage to the mucus layer, as described earlier; in addition, because these systems can diffuse readily through the mucus, they present the potential for “back-diffusion” back into the lumen, which could reduce the efficacy of the treatment. Since both methods have benefits and drawbacks that complement one another, systems that combine the methods (mucoadhesive and mucus-penetrating systems) may present the most promising path forward for system development.

Overall, these methods show promise in improving the efficacy of oral drug delivery. These methods demonstrate that knowledge of the structure, composition, and function of the mucus layers can be used to develop more effective oral drug delivery systems.

## Data Availability

Not applicable.
